# The complex immune puzzle: A deeper dive into the MORC1-mediated broad-spectrum defense signaling pathway

**DOI:** 10.1093/plcell/koaf075

**Published:** 2025-04-02

**Authors:** Meenu Singla-Rastogi

**Affiliations:** Assistant Features Editor, The Plant Cell, American Society of Plant Biologists; Department of Biology, Indiana University, Bloomington, IN 47405, USA

Plants have evolved a 2-layered immune system to combat environmental threats, including pathogen infection, herbivory, and physical damage associated with infection. The first layer, pattern-triggered immunity (PTI), is activated when surface-localized pattern recognition receptors detect molecular patterns derived from pathogens or the plant itself. Pathogens, however, have developed countermeasures by deploying effector proteins into host cells to suppress PTI (Peng et al. 2018). In response, plants have evolved a second layer of defense, effector-triggered immunity (ETI), mediated by nucleotide-binding domain leucine-rich repeat proteins that specifically recognize pathogen effectors (Cui et al. 2015). Both immune layers share key signaling components, including cytosolic calcium (Ca^2+^) influx, reactive oxygen species production, activation of mitogen-activated protein kinases, transcriptional reprogramming, and the production of defense hormones (Boudsocq et al. 2010; Peng et al. 2018). Ca^2+^ serves as a secondary messenger in plant immunity, playing a crucial role in initiating defense responses. Following immune activation, the cytosolic Ca^2+^ concentration increases, triggering calcium-dependent defense mechanisms. Calcium-dependent protein kinases (CDPKs or CPKs) contain Ca^2+^-binding motifs that enable them to sense subtle spatial and temporal Ca^2+^ oscillations and phosphorylate specific target proteins, thereby translating calcium signatures into specific defense responses (Boudsocq et al. 2010).

Among calcium-dependent protein kinases, CPK5 plays a pivotal role across multiple immune responses, including PTI, ETI, and systemic acquired resistance (SAR). This versatility stems from its ability to phosphorylate and activate critical transcription factors and defense-related proteins (Yip Delormel et al. 2022 ). One of the significant outcomes of this transcriptional activation is the induction of salicylic acid (SA) biosynthesis. Notably, both local and systemic resistance relies on the biosynthesis and accumulation of SA, which leads to the activation of downstream signaling pathways that are integral to PTI, ETI, and SAR (Zhang and Li 2019). Despite considerable advances in understanding CPK5's immunological functions, substantial knowledge gaps remain regarding its complete substrate repertoire and the precise signaling networks through which CPK5 orchestrates immune responses.

In new work, Congcong Sun, Yongming Chen, Aifang Ma, Pan Wang, and colleagues (Sun et al. 2025) identified MICRORCHIDIA 1 (MORC1), a member of the evolutionarily conserved GHKL-type ATPase family, as a substrate of CPK5. Previously, MORC1 was implicated—along with its close homolog MORC2—in multiple aspects of plant immunity, including PTI and ETI (Kang et al. 2008, 2010, 2012); however, the molecular mechanisms underlying these functions remained poorly understood. To elucidate the mechanism underlying MORC1-mediated plant immune regulation, the authors first screened for MORC1-interacting proteins by conducting liquid chromatography-tandem mass spectrometry analysis. Their analysis revealed CPK5 as a significant binding partner of MORC1, an interaction that they validated via split-luciferase complementation and bimolecular fluorescence complementation assays in *Nicotiana benthamiana*. Using co-immunoprecipitation assays, they demonstrated that the association between CPK5 and MORC1 was significantly enhanced by SA treatment, indicating that CPK5 interacts with MORC1 in response to activated immune signaling.

Both plant and human MORC proteins have been shown to exhibit DNA-modifying activities, though they require additional cofactors to achieve full functionality (Manohar et al. 2017). Given that DNA modification typically occurs within the nucleus, this raises a key question: how does cytosolic MORC1 achieve stabilization and nuclear translocation to execute its immune functions? Sun et al. (2025) address this question by demonstrating that CPK5 predominantly phosphorylates MORC1 at Thr122 residue. Through cell-free protein degradation experiments, they further establish that this site-specific phosphorylation is essential for MORC1 protein stability (Figure). Using cell fractionation and immunoblotting analyses, the authors then show that the phosphorylation-deficient MORC1 mutant failed to localize to the nucleus under both basal conditions and following SA treatment, suggesting phosphorylation is essential for promoting MORC1 nuclear translocation. Indeed, the level of nucleus-localized MORC1 protein has previously been shown to increase upon immune activation (Kang et al. 2012).

**Figure.**
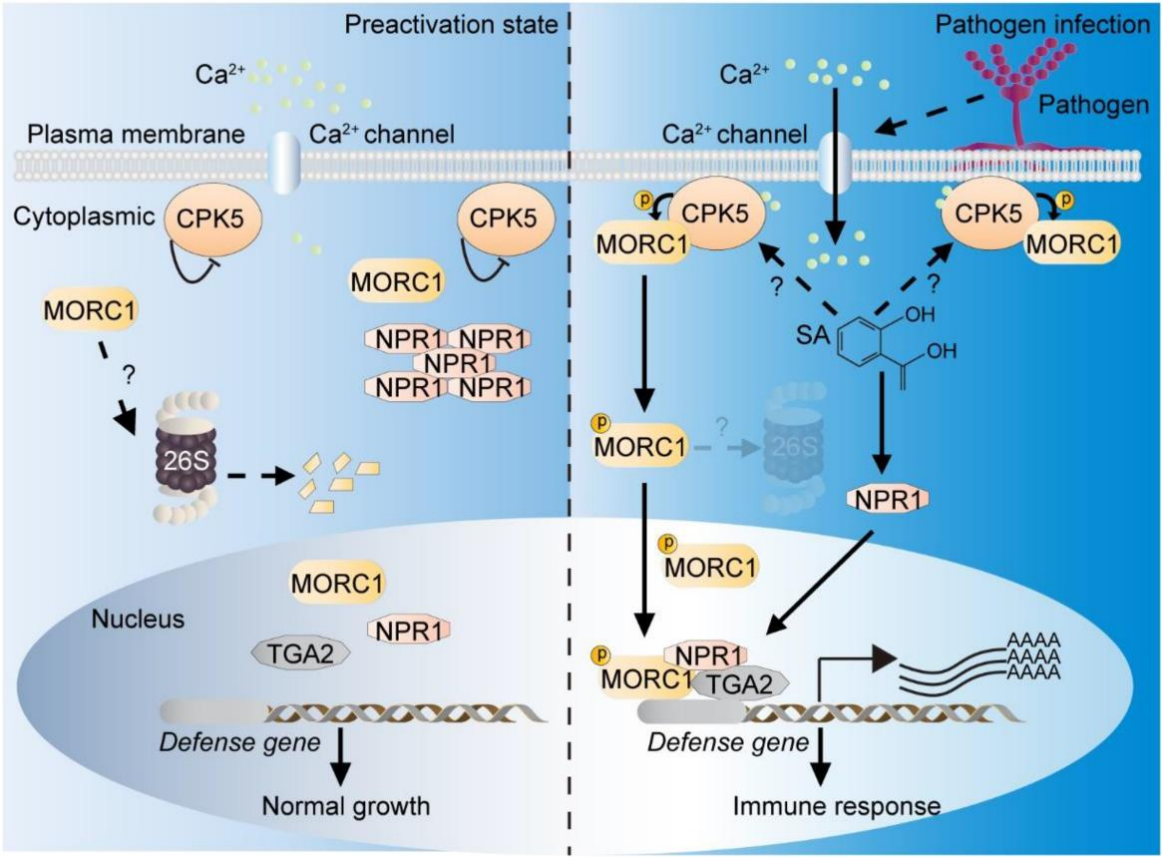
Working model for the CPK5-MORC1-NPR1-TGA module in pathogen-induced defense gene activation and disease resistance. Under normal conditions, MORC1 localizes to the cytosol, where it undergoes proteasome-mediated degradation via an unknown mechanism. Upon pathogen attack, a calcium channel opens, leading to a rapid Ca^2+^ influx in the cytosol, which activates CPK5. Activated CPK5 interacts with and phosphorylates MORC1, enhancing its stability and promotes its nuclear localization. Subsequently, nuclear-localized MORC1 associates with the NPR1-TGA complex, facilitating defense gene expression and disease resistance in Arabidopsis. Reprinted from Sun et al. (2025) Figure 8.

Once in the nucleus, MORC1 was shown to interact with NPR1 and TGA transcription factors through the split-luciferase complementation assays. The authors propose that this nuclear MORC1-NPR1-TGA complex functions as a regulatory hub to activate the expression of diverse defense-related genes. Furthermore, overexpression of MORC1 was shown to enhance SAR in plants, demonstrating improved defense capacity against a broad spectrum of pathogens. While this study has made significant strides in elucidating the role of MORC1 in immune signaling, further research is needed to decipher the precise molecular mechanisms through which the MORC1-NPR1-TGA complex regulates defense gene expression and coordinates immune induction.

This work elucidates a novel CPK5-MORC1-NPR1-TGA signaling module that orchestrates transcriptional activation of defense genes during plant immune responses (Figure). Using reverse genetic analyses, the authors found that MORC1 and MORC2 positively regulate broad-spectrum disease resistance against both biotrophic and necrotrophic pathogens in Arabidopsis, highlighting their essential role in plant immunity. These findings position MORC1 and its homologs as promising targets for engineering durable resistance in crop plants, potentially offering sustainable solutions to combat diverse pathogen threats in agriculture.

## Recent related articles in *The Plant Cell*

- Ren et al. (2024) reveal a mechanism through which MORC proteins, when recruited to 45S rDNA loci, regulate the expression of different 45S rRNA gene variants.
- Liu et al. (2024) show that CPK5 phosphorylation-induced CAMTA3 repressor destabilization triggers autoimmunity.
- Sun et al. (2022) decode the cooperative actions of calcium sensors in regulating plant immunity against soil-borne fungal pathogens.
- Zhou et al. (2020) describe the mechanism through which CPK5/CPK6 and MPK3/MPK6 cooperatively regulate camalexin biosynthesis upon pathogen infection.

## Data Availability

New data has not been generated or analyzed in support of this research.

## Dive Curated Terms

The following phenotypic, genotypic, and functional terms are of significance to the work described in this paper:

CPK5 Gramene: AT4G35310
CPK5 Araport: AT4G35310

NPR1 Gramene: AT1G64280
NPR1 Araport: AT1G64280

MORC1 Gramene: AT4G36290
MORC1 Araport: AT4G36290
